# The host sex contributes to the endophytic bacterial community in *Sargassum thunbergii* and their receptacles

**DOI:** 10.3389/fmicb.2024.1334918

**Published:** 2024-03-15

**Authors:** Yayun Zhao, Tao Sun, Yang Li, Zhibo Yang, Jun Chen, Jing Wang, Xinlong Yu, Xuexi Tang, Hui Xiao

**Affiliations:** ^1^College of Marine Life Sciences, Ocean University of China, Qingdao, China; ^2^Laboratory for Marine Ecology and Environmental Science, Qingdao Marine Science and Technology Center, Qingdao, China; ^3^Qingdao Branch CCCC Water Transportation Consultants Co.,LTD, Qingdao, China; ^4^Shandong Marine Forecast and Hazard Mitigation Service, Qingdao, China

**Keywords:** endophytic bacteria, host sex, macroalgae, *Sargassum thunbergii*, receptacles

## Abstract

Endophytic bacteria have a complex coevolutionary relationship with their host macroalgae. Dioecious macroalgae are important producers in marine ecosystems, but there is still a lack of research on how sex influences their endophytic bacteria. In this study, the endophytic bacterial communities in male and female *S. thunbergii* and their reproductive tissues (receptacles) were compared using culture methods and high-throughput sequencing. The endophytic bacterial communities detected by the two methods were different. Among the 78 isolated strains, the dominant phylum, genus, and species were *Bacillota*, *Alkalihalobacillus*, and *Alkalihalobacillus algicola*, respectively, in the algal bodies, while in the receptacles, they were *Bacillota*, *Vibrio*, and *Vibrio alginolyticus*. However, 24 phyla and 349 genera of endophytic bacteria were identified by high-throughput sequencing, and the dominant phylum and genus were *Pseudomonadota* and *Sva0996_ Marine_ Group*, respectively, in both the algal body and the receptacles. The two methods showed similar compositions of endophytic bacterial communities between the samples of different sexes, but the relative abundances of dominant and specific taxa were different. The high-throughput sequencing results showed more clearly that the sex of the host alga had an effect on its endophyte community assembly and a greater effect on the endophytic bacterial community in the receptacles. Moreover, most specific bacteria and predicted functional genes that differed between the samples from the males and females were related to metabolism, suggesting that metabolic differences are the main causes of sex differences in the endophytic bacterial community. Our research is the first to show that host sex contributes to the composition of endophytic bacterial communities in dioecious marine macroalgae. The results enrich the database of endophytic bacteria of dioecious marine macroalgae and pave the way for better understanding the assembly mechanism of the endophytic bacterial community of algae.

## 1 Introduction

Endophytic bacteria are commonly present in plants and coevolve with their hosts, playing an important role in host growth, development and disease resistance. Studying the community structure of endophytic bacteria in plants is necessary for analyzing the functions of these bacteria and elucidating endophyte-host interactions.

Macroalgae are an important component of marine ecosystems and play a crucial role in material cycling and energy flow. At present, research on the community structure of endophytic bacteria in plants mainly focuses on higher terrestrial plants, and much work has been done on isolating, identifying, and describing the physiological and biochemical characteristics of endophytic bacteria, as well as uncovering their interactions with hosts (Afzal et al., 2019; Ling et al., 2022; Yarte et al., 2022). In recent years, high-throughput sequencing technology has also been widely used to obtain a large amount of information on the endophytic bacterial community in terrestrial plants, includingthe crops *Oryza sativa* (Liu Y. et al., 2019), *Zea mays* (Liu Y. et al., 2020) and the medicinal plants *Panax notoginseng* (Zhang C. et al., 2020) and *Gastrodia elata f. glauca* (Zheng et al., 2022), as well as some aquatic plants, such as Lemnaceae, *Pontederia crassipes*, *Pistia* (Pramanic et al., 2023), and *Glehnia littoralis* (Huo et al., 2020). There have also been some studies on isolating, identifying, and describing the physiological activity and function of algal endophytic bacteria (Song et al., 2015; Zhang, 2017; Feng et al., 2020; Amri et al., 2023) based on culture methods. However, Hollants et al. (2011) used the denaturing gradient gel electrophoresis (DGGE) method to study the endophytic bacterial community of *Bryopsidales* (Chlorophyta), and Mei (2019) applied high-throughput sequencing to investigate the endophytic bacterial community in *Sargassum horneri* and *Ulva prolifera*, but there have been no studies on the differences in endophytic bacteria between the different sexes of algae.

There are many factors that can affect the composition of endophytic bacterial communities, but relevant studies have mainly been carried out on land plants. Previous studies have shown that both the external environment and internal factors influence plant endophytic bacterial composition (Afzal et al., 2019). The number and species of endophytic bacteria have different distributions in plants of different habitats (Zhang A. et al., 2020). The reason why the external environment can influence bacterial composition is that bacterial metabolic function changes depending on soil nutrients, contaminants and temperature, which lead to a strong selection effect on the bacterial population (Zhang et al., 2015). On the other hand, internal factors such as host plant species, tissue location and life history stage all have effects on the endophytic bacterial community (Dastogeer et al., 2018; Mei et al., 2021). The vertical transmission of endophytic bacteria enables these bacteria to be transferred between generations of plants and form a stable symbiotic relationship with plants. Endophytic bacteria differ among different host plants (Liaqat and Eltem, 2016), different varieties of the same plant species (Munir et al., 2020) and even among different seed genotypes (Liu Y. et al., 2020). There are also differences in endophytic bacterial communities among different tissues of the same plant, as confirmed by high-throughput sequencing in *Arachis hypogaea Linn* (Li et al., 2021), *Zea mays L*. (Marag and Suman, 2018), and *Hippophae tibetana* (Zhang A. et al., 2021). Additionally, at different growth stages of the plant, the endophytic bacterial community will undergo corresponding changes due to alterations in the internal and external environments (Mahmood et al., 2019; Song et al., 2020).

However, host sex is rarely mentioned among the factors affecting the community structure of endophytic bacteria. In fact, sex can lead to differences in the morphology, structure and function of dioecious plants (Tang, 2020; Lu et al., 2021; Zeng et al., 2022), and there are also some differences in enzyme activity (Chen et al., 2020), secondary metabolites (Li et al., 2022) and endogenous hormone levels (Ge et al., 2021), but there have been few studies on sex differences in endophytic bacteria in plants. Some studies have revealed differences in epiphytic bacterial communities in plants of different sexes, such as in *Populus cathayana* (Liu et al., 2021), *Sargassum thunbergii* (Wang et al., 2022b), and *Porphyra haitanensis* (Yang et al., 2022). However, for marine macroalgae, whether sex has an impact on the assembly of endophytic bacterial communities is still unknown.

*Sargassum thunbergii* is a common intertidal macroalgae in coastal China. As the feed of valuable aquaculture organisms and the preferred plants for marine pastures, there is an urgent need for artificial cultivation of *S. thunbergii*. Endophytic bacteria are closely related to the growth and development of *S. thunbergii*, and their study will facilitate its cultivation. *S. thunbergii* is a typical dioecious alga with receptacles appearing in the reproduction stage (Wang, 2007). There are obvious differences in the appearance and internal structure of female and male receptacles, and they perform different reproductive functions (Wang et al., 2007). Whether these differences will lead to differences in the endophytic bacterial communities between male and female *S. thunbergii* and their receptacles and whether host algal sex will affect the endophytic bacterial community have not yet been explored.

In this study, the endophytic bacterial community was compared between males and females of the intertidal macroalga *S. thunbergii* and their receptacles from Shandong Peninsula by culture-dependent and high-throughput sequencing technologies, aiming at enriching the basic information on the endophytic bacterial community in marine macroalgae and elucidating the role of host sex in the assembly of the endophytic bacterial community in *S. thunbergii*.

## 2 Materials and methods

### 2.1 Sample collection

The algal samples were collected from a 5 m × 100 m sampling square in the continuous intertidal sea area at Taipingjiao (120°21′34.2″E, 36°14′58.3″N) along the coast of Qingdao (China) on July 21, 2021, during the reproductive period of *S. thunbergii*, and then placed in sterile sample bags and brought back to the laboratory for further processing within 30 min. The sex of the algae was confirmed in the laboratory by observing the internal structure of the receptacles using a microscope (Nikon H600L, Tokyo, Japan).

### 2.2 Culture-dependent processing

For the culture dependent test, six strains of *S. thunbergii* (3 males and 3 females) were used and these samples were from 3 different sampling sties, respectively. The surface of the algae was disinfected on a clean bench by a method established in the pre-experiment (75% alcohol for 5 min + 2.5% sodium hypochlorite for 10 min) and finally washed with sterile water 7 times. Then, 0.1 mL of the final sterile water rinse was collected and spread on Zobell 2216E medium plates. When cultured in a biochemistry incubator (SPX-150, Wanfeng Instrument Co. Ltd., China) at 25°C for 48 h, if no colonies had grown, the surface disinfection of the algae was considered successful.

On the clean bench, 2 g of male and female body tissues and their receptacles (picked with sterile tweezers were taken from disinfected *S. thunbergii* individuals and transferred to a sterile mortar). Eight milliliters of sterile seawater were added to grind the samples into a homogenized suspension. The dilution 10^0^, 10^–1^, and 10^–2^ were prepared and 0.1 mL of the suspension was spread on Zobell 2216E medium plate with three replicates. Then, the plates were placed in a biochemical incubator at 25°C for 48 h. Since the number of colonies of dilution 10^0^ group was less than thirty while that of dilution 10^–1^ group was less five and that of dilution 10^–2^ group was zero, all colonies grown on the plates of dilution 10^0^ group were isolated and purified by the continuous streaking method, and the obtained strains were stored in stroke-physiological saline solution containing 15% glycerol at −80°C.

Bacterial DNA was extracted from the isolated strains using a TIANamp Bacterial DNA Kit [Tiangen Biotech (Beijing) Co., Ltd.]. The 16S rDNA sequence was then amplified using the forward primer 27F and reverse primer 1492R, after that sequencing was conducted by Sangon Biotech (Shanghai) Co., Ltd. The sequencing results were spliced and compared with the EzBioCloud database.^1^ The top 10 strains with the closest similarity among the strains with more than 98% similarity were selected. A phylogenetic tree was constructed with the sequences through three methods with MEGA 11.0 software, namely, neighbor-joining (NJ), maximum likelihood (ML) and minimum-evolution (ME), to determine the species of the strains.

### 2.3 High-throughput sequencing and sequence processing

On the clean bench, 2 g of male and female body tissues and receptacles (picked with sterile tweezers in an ice bath) was taken from the disinfected *S. thunbergii* individuals, transferred to a sterile sample tube and stored at −80°C. DNA extraction and sequencing were performed by Guangzhou Kidio Biotechnology Co., Ltd. The samples were labeled Male-ENDO (endophytic bacteria in the male algal body), Female-ENDO (endophytic bacteria in the female algal body), M-ENDO-Receptacles (endophytic bacteria in male receptacles) and F-ENDO-Receptacles (endophytic bacteria in female receptacles). Each group had 8 replicates and each sample came from one alga.

After genomic DNA was extracted from the samples, the V3 + V4 region of 16S rDNA was amplified with a specific primer with a barcode. The primer sequences were 341F: CCTACGGGNGGCWGCAG and 806R: GGACTACHVGGG TATCTAAT. Then, the PCR amplification products were cut and recovered, the purified amplification products were mixed in equal amounts, the sequencing joints were connected, the sequencing library was constructed, and high-throughput sequencing was performed on the Illumina NovaSeq 6000 PE250 platform (Illumina, San Diego, CA, USA).

### 2.4 Data analysis

UPARSE software (version 9.2.64_i86linux32) was used to concatenate and deduplicate sequences, sequences with more than 97% similarity were clustered into an operational taxonomic unit (OUT), and the SILVA (version 132) database was used to classify the OTUs. The Chao1, Ace, Shannon and Simpson α-diversity indices were calculated by QIIME (version 1.9.1.) The significance of intergroup index comparisons was determined by the Kruskal-Wallis (KW) test and Welch’s *t*-test. β-diversity was analyzed using principal coordinate analysis (PCoA) and the unweighted pair-group method using arithmetic averages (UPGMA) approach based on Bray-Curtis distances. According to the OTU classification, the high-throughput sequencing data were used to cluster the bacteria at the phylum and genus levels, the species not clearly classified or with relative abundance less than 1% were classified as “others,” and a histogram was drawn. Linear discriminant analysis effect size (LEfSe) software was used to analyze the differences between groups. The KW rank sum test was performed among samples from all groups first, and then the Wilcoxon rank sum test was used to compare the selected species between the two groups. Linear discriminant analysis (LDA) was used to sort the selection results and generate the LDA difference analysis diagram, and then an evolutionary branching diagram was obtained by mapping the differences onto the classification tree with a known hierarchical structure. Finally, PICRUSt (version 2.1.4) was used to predict the function of endophytic bacteria, and the KW test was used to analyze the significance of functional differences.

### 2.5 Data availability

The bacterial sequences obtained in this study have been saved to the National Center for Biotechnology Information (NCBI) with BioProject IDs: PRJNA830829 and PRJNA830307.

## 3 Results

### 3.1 Culturable endophytic bacteria

In this study, a total of 78 bacterial strains were isolated from male and female *S. thunbergii* and their receptacles and their phylogenetic tree is shown in Supplementary Figure 1. Additionally, the functions of isolated strains reported in previous studies were listed in Supplementary Table 1.

**FIGURE 1:**
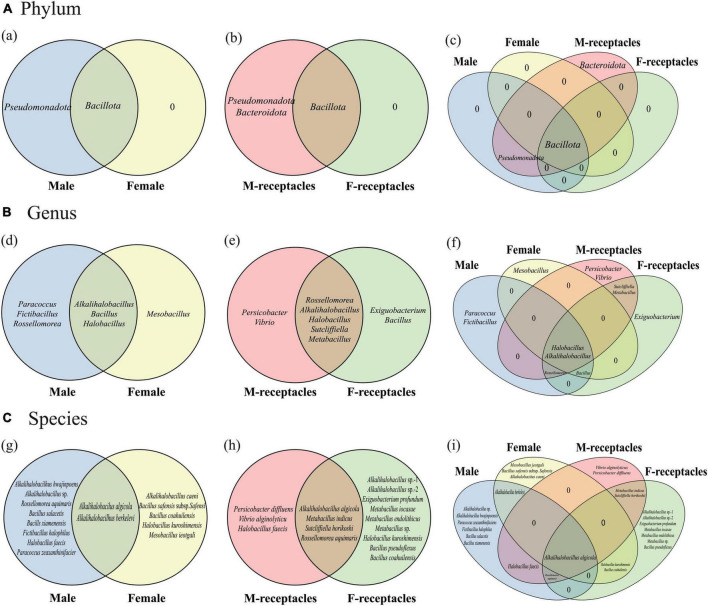
Venn diagrams of culturable heterotrophic bacteria of male and female *S. thunbergii* and their receptacles. **(A)** Phylum level. **(B)** Genus level. **(C)** Species level.

Thirty-one bacterial strains were isolated from algal bodies (19 from males and 12 from females), which belonged to 2 phyla, 7 genera, 14 species, and 1 suspected new species (Supplementary Table 1A). The dominant phylum, genus, and species were *Bacillota*, *Alkalihalobacillus* and *Alkalihalobacillus algicola*, respectively. In addition, 1 phylum (*Bacillota*), 3 genera (e.g., *Alkalihalobacillu*s), and 2 species (*A. algicola* and *A. berkeleyi*) were detected in the algal bodies of both sexes. However, 1 phylum (*Pseudomonadota*), 3 genera (e.g., *Rossellomorea*), and 8 species (e.g., *A. hwajinpoensis*) were isolated only from the male algal body, while 1 genus (*Mesobacillus*) and five species (e.g., *A. caeni*) were isolated only from the female algal body (Figure 1).

The culturable endophytic bacteria in receptacles were more diverse than those in algal bodies. Forty-seven strains (21 from males and 26 from females) belonging to 3 phyla, 9 genera, 13 species (Supplementary Table 1B), and 3 suspected new species were also isolated. One phylum (*Bacteroidota*) and five genera (e.g., *Exiguobacterium*) were isolated only from the receptacles (Figure 1), and the dominant phylum was the same as in the algal body, but the dominant genus and species changed to *Vibrio* and *V. alginolyticus*, respectively. In addition, various samples shared 1 phylum (*Bacillota*), 2 genera (*Alkalihalobacillus* and *Halobacillus*) and 1 species (*A. algicola*), but each kind of sample had specific bacteria (Figure 1).

Moreover, the culturable endophytic bacteria isolated from the receptacles were very different between the sexes. The dominant phylum, genus and species in the male receptacles were *Pseudomonadota*, *Vibrio*, and *V. alginolyticus*, while in the female receptacles, they were *Bacillota*, *Alkalihalobacillus*, *Metabacillus*, and *E. profundum*, respectively. One phylum (*Bacillota*), 5 genera (e.g., *Alkalihalobacillus*), and 4 species (e.g., *A. algicola*) were shared by male and female receptacles. However, many bacterial taxa could be isolated only from the receptacles of one sex. For example, *Pseudomonadota* and *Bacteroidota* were specific to male receptacles, while there were no specific phyla in female receptacles. Additionally, there were two genera (*Vibrio* and *Persicobacter*) and three species (e.g., *V. alginolyticus*) specific to male receptacles, while two genera (*Exiguobacterium* and *Bacillus*) and nine species (such as *E. profundum*) were specific to female receptacles.

### 3.2 High-throughput sequencing results

Because the DNA extracted from one Male-ENDO sample was unqualified, the results of 31 samples were ultimately analyzed, and a total of 3,377,830 sequences were obtained. After mass filtering and removal of chimeric, chloroplast and mitochondrial sequences, 3,195,870 optimized sequences were obtained. The coverage of all samples was above 99% (Supplementary Figure 1), indicating that the sequencing depth covered most of the bacteria in the samples and the sequencing data were reliable and effective.

#### 3.2.1 α–diversity

**FIGURE 2:**
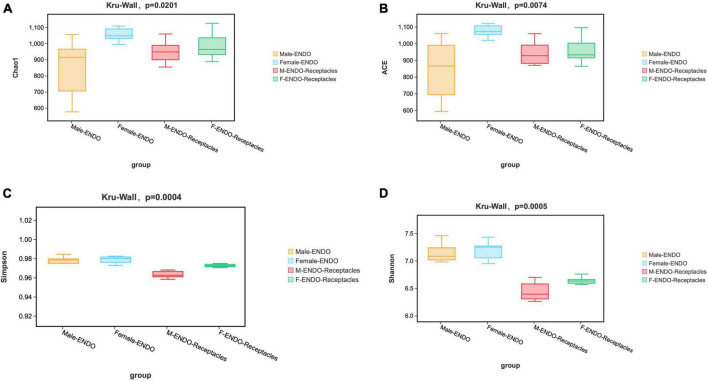
The α-diversity of endophytic bacterial communities in male and female *S. thunbergii* and their receptacles. **(A)** Chao1 index. **(B)** ACE index. **(C)** Simpson index. **(D)** Shannon index.

The results of α-diversity analysis (Figure 2) revealed significant differences in the four indices among the four groups of samples (KW test, *P* < 0.05). Both the Chao1 and Ace index results showed that the abundance of bacteria in female algae was the highest, followed by that in female receptacles, that in male receptacles and that in male algal bodies. The Shannon and Simpson indices showed that the bacterial diversity in the algal body was higher than that in the receptacle. Interestingly, the four indices were higher in females than in males, indicating that the abundance and diversity of endophytic bacteria in the female samples were higher than those in the male samples.

#### 3.2.2 β–diversity

**FIGURE 3:**
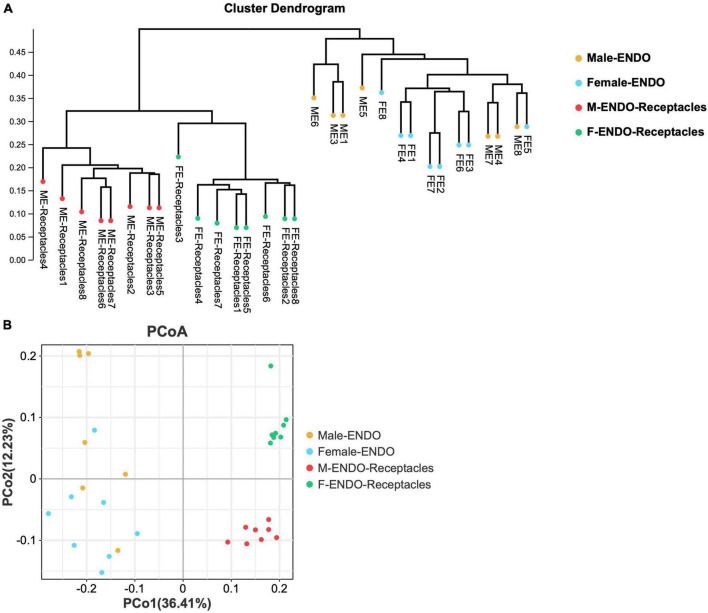
The β-diversity of endophytic bacterial communities in male and female *S. thunbergii* and receptacles. **(A)** Results of the unweighted pair-group method with arithmetic mean (UPGMA) approach. **(B)** Results of principal coordinate analysis (PCoA).

The results of UPGMA and PCoA based on Bray-Curtis distances (Figure 3) clustered the bacteria from male and female *S. thunbergii* and their receptacles, indicating that the samples in each group were similar, but the differences between groups were significant (*P* < 0.01). Notably, the clustering of endophytic bacteria in male and female receptacles was more obvious than that in algal bodies, indicating that the differences between male and female receptacles were larger than those between male and female algal bodies.

#### 3.2.3 Shared bacteria and specific bacteria

**FIGURE 4:**
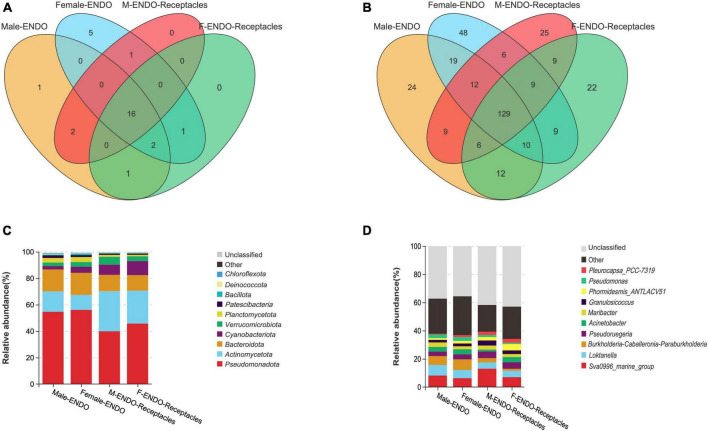
Venn diagram and relative abundance of endophytic bacteria in algal bodies and receptacles of male and female *S. thunbergii.* **(A,C)** Phylum level. **(B,D)** Genus level.

The Venn diagram (Figure 4) showed that the shared bacterial taxa accounted for the majority in the four kinds of samples. There were 16 shared endophytic bacteria at the phylum level (Figure 4A), and the core phyla (with relative abundances higher than 1%) included *Pseudomonadota* (39.89–56.03%), *Bacteroidota* (11.73–16.70%), *Actinomycetota* (11.37–30.31%), *Planctomycetota* (1.13–3.75%), *Verrucomicrobiota* (2.68–5.97%), and *Cyanobacteria* (2.57–10.52%). At the genus level (Figure 4B), there were 129 shared genera, and the core genera (with relative abundances higher than 1%) included *Sva0996_ Marine_ Group* (6.27–13.01%), *Loktanella* (4.44–7.46%), *Burkholderia Caballeronia Paraburkholderia* (1.39–7.51%), *Acinetobacter* (1.35–3.66%), *Mariactor (*2.12–3.23%), *Pseudoruegeria* (3.19–4.70%), *Granulosicoccus* (1.81–3.62%), and *Phormidesmis_ ANTLACV51* (1.23–4.50%).

Each kind of sample also had specific endophytic bacterial taxa. For instance, there were 5 specific phyla (e.g., *Entotheonellaeota* and *Halanaerobiaeota*) in female algal bodies and only one specific phylum (Elusimicrobiota) in male algal bodies, but there were no specific phyla in the receptacles. There were 24 genera specific to male algal bodies, including *Brachybacterium*, *Oikopleura_dioica*, and *Magnetospira*, while 48 genera were specific to female algal bodies, including *Macrococcus*, *Candidatus_Actinomarina*, and *Marinobacterium*. For the receptacles, there were 25 specific genera in males (e.g., *Prevotella_1*, *Muricauda*, and *Erysipelotrichaceae_UCG-007*), while 22 genera were specific to female receptacles (e.g., *Shewanella*, *Minicystis*, and *Steroidobacter*).

#### 3.2.4 Community composition and dominant endophytic bacteria

The community composition and relative abundance of the endophytic bacteria in male and female *S. thunbergii* and their receptacles are shown in Figure 4. The compositions were similar among the four kinds of samples, but the abundance of some bacterial taxa differed obviously. At the phylum level, the top three phyla in algal bodies were *Pseudomonadota*, *Bacteroidota*, and *Actinomycetota*, with relative abundances of 54.62, 16.58, and 15.42% in male algal bodies and 56.03, 16.70, and 11.37% in female bodies, respectively. In receptacles, the top three phyla were the same, but the order changed to *Pseudomonadota*, *Actinomycetota*, and *Bacteroidota*, and the abundance of the top three phyla was 39.89, 30.31, and 12.35% in males and 45.72, 24.93, and 11.73% in females, respectively. Notably, *Cyanobacteria* was ranked fourth in female algal bodies and the receptacles of both sexes but was ranked sixth in male algal bodies.

Additionally, the dominant genera of endophytic bacteria in the four kinds of samples were basically the same but differed in relative abundance. The top three dominant genera in male algal bodies were *Sva0996_marine_group* (8.16%). *Loktanella* (7.46%) and *Burkholderia-Caballeronia-Paraburkholderia* (6.37%), while they were *Burkholderia-Caballeronia-Paraburkholderia* (7.51%), *Sva0996_marine_group* (6.27%), and *Loktanella (*5.82%) in female algal bodies. The top three dominant genera in male and female receptacles were the same, namely, *Sva0996_marine_group* (males: 13.01%; females: 7.00%), *Pseudoruegeria* (males: 4.70%; females: 4.70%), and *Loktanella* (males: 4.44%; females: 4.53%).

#### 3.2.5 Network analysis between bacterial community

**FIGURE 5:**
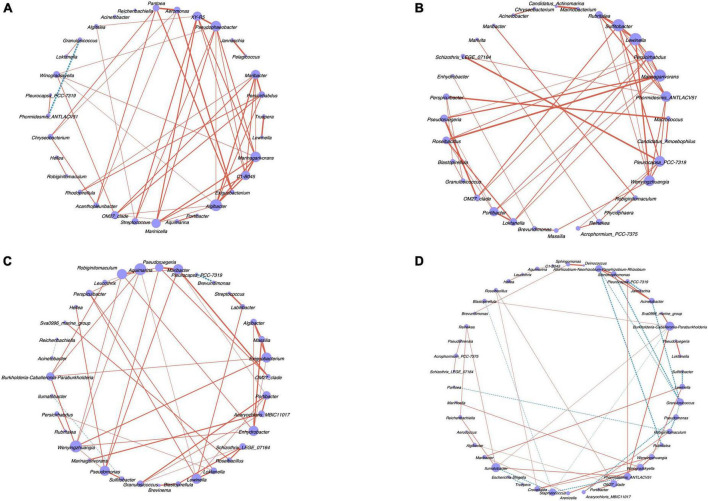
The network analysis of endophytic bacteria in algal bodies and receptacles of male and female *S. thunbergia* at genus level (*P* < 0.05). **(A)** Male algal bodies. **(B)** Female algal bodies. **(C)** Male receptacles. **(D)** Female receptacles. Node color corresponds to genus taxonomic classification. Edge color represents positive (red) and negative (blue) correlations.

The network analysis (Figure 5) based on the relationship pairs with the top-50 correlation coefficient showed the specific network of the endophytic bacterial community in male and female algal bodies and receptacles of *S. thunbergia*. The results indicated that the correlation between the endophytic bacteria in four kinds of samples was significantly different (*P* < 0.05). The node number of endophytic bacteria at genus level in female algal bodies were larger than in male algal bodies, while the results in receptacles was opposite.

Compared to the interaction between the endophytes in different sex algal bodies through the network analysis, 50 nodes and 1225 edges in the microbial network from *S. thunbergia* were found. Also, the endophytes in female *S. thunbergia* had 56 nodes and 1540 edges. *Algibacter*, *Marinagarivorans*, *Pseudophaeobacter*, and *Maribacter* showed high betweenness in male algal bodies, which indicated that these microbes were the key nodes of this sample group. The key nodes of the endophytes in female algal bodies were *Sulfitobacter*, *Marinagarivorans*, *Lewinella*, and *Phormidesmis_ANTLACV51* with a high level of betweenness.

The interaction between the endophytes in male and female receptacles of *S. thunbergia* was also different. The microbial network from female receptacles had 40 nodes and 780 edges, while that from male receptacles had 54 nodes and 1431 edges. In the microbiome of male receptacles from *S. thunbergia*, *Aquimarina*, *Wenyingzhuangia*, *Maribacter*, and *Pseudomonas* showed high betweenness centrality in the community network, while *Burkholderia-Caballeronia-Paraburkholderia*, *Ilumatobacter*, *Phormidesmis_ANTLACV51*, and *Granulosicoccus* were the key nodes of the endophytic microbial community in female receptacles.

#### 3.2.6 Biomarkers

**FIGURE 6:**
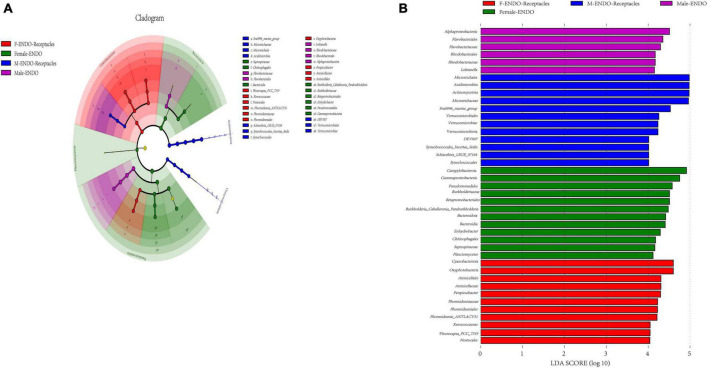
Biomarker analysis of male and female *S. thunbergii* and their receptacles. The cladogram shows the phylogenetic structure of the microbiota. The circles that radiate from inside to outside represent taxonomic levels from phylum to genus, and each small circle represents an individual taxon. The diameter of the circles is proportional to the relative abundance. The linear discriminant analysis (LDA) scores indicate significant differences in bacterial taxa (LDA score >4.0). **(A)** Cladogram. **(B)** LDA score chart.

The LEfSe analysis (Figure 6) revealed the endophytic bacterial taxa with significant differences between groups (LDA > 4), namely, biomarkers, and the results indicated that there were many biomarkers between the four kinds of samples. *Alphaproteobacteria* (class), *Flavobacteriales* (order), and *Flavobacteriaceae* (family) were enriched in male algal bodies, while *Pseudomonadota* (phylum), *Gammaproteobacteria* (class), and *Pseudomonadales* (order) were enriched in female algal bodies; *Microtrichales* (order), *Acidimicrobiia* (class), and *Actinomycetota* (phylum) were abundant in male receptacles, while *Cyanobacteria* (phylum), *Oxyphotobacteria* (class), and *Arenicellaceae* (family) were abundant in female receptacles.

**FIGURE 7:**
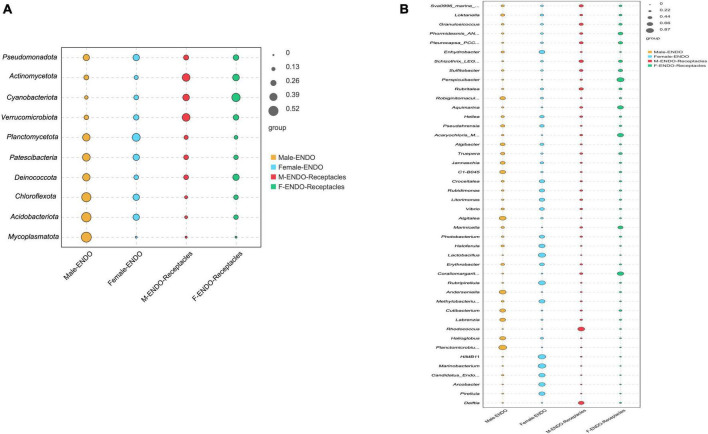
Indicator value analysis of endophytic bacteria in male and female *S. thunbergii* and their receptacles at the phylum and genus levels (*P* < 0.01). **(A)** Phylum level. **(B)** Genus level.

In addition, Figures 7A, B. showed the indicator values of the groups with significant differences (*P* < 0.01) in abundance between male and female *S. thunbergii* and their receptacles at the phylum and genus levels. The groups showed large differences in indicator values. Among the indicator species at the genus level, *Planctomicrobium*, *Anderseniella*, and *Algitalea* were abundant in the male algal bodies; *HIMB11*, *Lactobacillus*, and *Marinobacterium* were enriched in the female algal bodies; *Rhodococcus* and *Delftia* were abundant in the male receptacles; and *Perspicuibacter*, *Aquimarina*, and *Coraliomargarita* were enriched in the female receptacles. This suggests that the highest frequency or abundance of endophytic bacteria was not the same between the algal body and the receptacles or between samples of different sexes.

#### 3.2.7 Prediction of functional genes of endophytic bacteria

**FIGURE 8:**
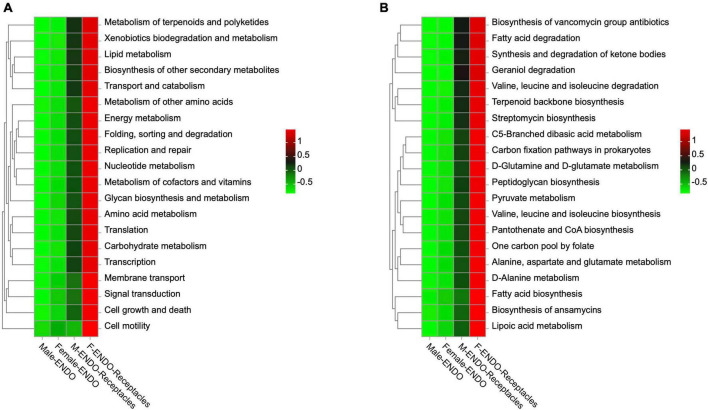
Functional prediction of genes of endophytic bacteria in male and female *S. thunbergii* and their receptacles. **(A)** Level 2. *P* < 0.01. **(B)** Level 3. *P* < 0.05 (KW rank sum test).

Based on PICRUSt2, the gene functional abundance of the endophytic bacteria in male and female *S. thunbergii* and their receptacles was predicted. The results showed significant differences among the 4 groups of samples (Figure 8). At the secondary level, there was a significant difference (*P* < 0.01) in the function of 27 out of 33 genes among the 4 kinds of samples. These functions included five categories: metabolism, genetic information processing, cellular processes, organismal systems, and environmental information processing. At the third level, there was a significant difference (*P* < 0.05) among the four kinds of samples in 151 out of 169 gene functions. Among them, 109 out of 151 gene functions were related to metabolism, such as xenobiotic biodegradation and metabolism (18 types), carbohydrate metabolism (15 types), amino acid metabolism (13 types), and lipid metabolism (12 types), and others included environmental adaptability, infectious diseases, and cell motility. Another interesting finding was that the abundance of predicted genes with significant differences in female receptacles was higher than that in males, but in the algal bodies of the two sexes, the abundances of predicted genes with significant differences were basically the same, and both were lower than those in the receptacles.

## 4 Discussion

### 4.1 Composition of the endophytic bacterial community in *S. thunbergia*

The results of both culture and high-throughput sequencing in this study showed that the endophytic bacterial species were very rich in *S. thunbergii* and their receptacles. Among the strains isolated from algal bodies, *Bacillota* dominated at the phylum level, which was consistent with the results of studies on terrestrial plants (Webster et al., 2020). However, the results at the genus level and species were different from those in terrestrial plants, with most of the detected bacteria being saline-alkali tolerant or salt tolerant bacteria (Supplementary Table 1), such as *A. algicola* (Ivanova et al., 2004), *A. berkeleyi* (Patel and Gupta, 2020), *A. caeni* (Patel and Gupta, 2020), *R. aquimaris* (Wang et al., 2023), *F. halophilus* (Sharma et al., 2016), *H. faecis* (Zhang et al., 2019), and *H. kuroshimensis* (Shi et al., 2020), indicating the adaptation of algal endophytic bacteria to the marine environment. It was interesting that the dominant species was *A. algicola*, which was an algae-dweller with alginolytic abilities and a lot of other isolated endophytic bacteria such as *H. kuroshimensis* (Shi et al., 2020), *V. alginolyticus* (de Souza Valente and Wan, 2021), and *P. diffluens* (Nikolaeva et al., 1999) were also alginic acid-dissolving bacteria. In addition, there were many bacterial species involved in material metabolism, included *P. caeni* (Baker et al., 1998) with multiple metabolic functions, *M. jeotgali* (Green-Ruiz et al., 2008) and *S. horikoshii* (Gupta et al., 2020) related to heavy metal absorption, etc. These results indicated that the metabolic relationship between endophytes and hosts, especially the metabolism of algal derived substances by endophytes, which was very important in the relationship between endophytes and hosts. Moreover, there are also some beneficial endophytic bacteria which were beneficial to the host, including antibacterial *B. Safensis* (Zhang Z. et al., 2020) and *B. xiamenensis* (Amna et al., 2020), as well as *M. indicus* (Falkenberg et al., 2023), which induced host metamorphosis, indicating that there was a very complex relationship between endophytic bacteria and host algae.

In addition, our study revealed that endophytic bacteria were more abundant in the receptacles than in the algal bodies, with five genera isolated only in the receptacles. These genera were rich in functions; for example, *Exiguobacterium*, which is adapted to extreme environmental conditions such as salt and alkali (He et al., 2012; Zhang et al., 2013), the biodegrading bacterium *Persicobacter* (Han et al., 2012), and *Vibrio* (de Souza Valente and Wan, 2021), which is a common pathogen in marine organisms.

The high-throughput sequencing results showed that the core phyla of endophytic bacteria in *S. thunbergii* were *Pseudomonadota*, *Bacteroidota*, and *Actinomycetota*, which were similar to those in terrestrial plants (Fadiji et al., 2020) and rice seeds (Feng et al., 2023). However, there was a significant difference in the core genera of endophytic bacteria in *S. thunbergii* compared with terrestrial plants and the other macroalgae *S. horneri* and *U. prolifera* (Mei, 2019). This indicated that the species specificity of host algae is an important factor determining the composition of endophytic bacterial communities. The core endophytic bacteria in *S. thunbergii* included the genus *Sva0996_marine_group*, which is related to the metabolism of alga-derived substances (Orsi et al., 2016), the dimethylsulfoniopropionate (DMSP) degrader *Loktanella* (Sun et al., 2020), and pollutant-degrading bacteria, such as the phenanthrene (PHE)-degrading bacterium *Acinetobacter* (Li et al., 2017), the polyaromatic hydrocarbon (PAH)-degrading bacterium *Pseudoruegeria* (Yuan, 2008), and the hydrocarbon-degrading bacteria *Granulosicoccus* (Rizzo et al., 2019) and *Robiginitomaculum* (Verhoeven et al., 2017). In addition, there were some bacteria that can promote and inhibit the growth of algae, such as *Burkholderia-Caballeronia-Paraburkholderia* (Wu et al., 2022), *Enhydrobacter* (Dartora et al., 2016), and *Maribacte*r (Ghaderiardakani et al., 2019). This indicated that the composition of endophytic bacteria was closely related to the external environment and host alga. Interestingly, these endophytic bacteria were also abundant in *S. thunbergii* (Wang et al., 2022a), which is consistent with the findings of studies on *S. horneri* and *U. prolifera* (Mei, 2019), indicating that the endophytic bacteria and epiphytic bacteria in macroalgae have a common source and close connection.

### 4.2 The differences in endophytic bacterial communities between male and female *S. thunbergii* and their receptacles

Previous studies paid little attention to the differences in endophytic bacterial communities between hosts of different sexes, and similar studies have not been carried out in macroalgae. However, the results of both the culture method and high-throughput sequencing in this study revealed differences between endophytic bacteria in algal bodies and receptacles of hosts of different sexes.

The results of both methods showed a difference in the endophytic bacterial community between male and female *S. thunbergii* and their receptacles. Although the dominant bacteria of female and male algal bodies were basically the same, some species could be isolated from only a single sex. Interestingly, some endophytes isolated from algae of different sexes, although not the same species, performed similar functions. For example, *H. trueperi* with antibacterial activity was isolated from male algae (Chen et al., 2010), while *B. safensis* (Zhang Z. et al., 2020), which were isolated from female algal bodies, also have antibacterial properties. There have been many relevant studies on endophytic bacteria isolated from different plant organs (Wang et al., 2021; Zhang A. et al., 2021). However, the differences in culturable bacteria among different reproductive tissues of dioecious plants have not been reported. This study revealed that the dominant endophytic bacteria differed between the male and female receptacles of *S. thunbergia.* For example, *V. neocaledonicus*, the dominant bacterium isolated only from the male receptacles, has been reported as a pathogen and has the ability to prevent oxygen from entering rusted biofilm (Moradi et al., 2018). The dominant genus *Alkalihalobacillus* in female receptacles has a degradation function (Song et al., 2023).

The results of high-throughput sequencing of the specific taxa more clearly showed the differences in endophytic bacterial communities between sexes. The endophytic bacteria of samples from different sexes included specific phyla and genera, and most of them were metabolism-related bacteria. For example, among the specific bacterial phyla in the female algal bodies, *Entotheonellaeota* has the ability to synthesize bioactive substances (Ho et al., 2021), Armatimonadota and *Planctomycetota* can decompose and utilize complex polysaccharides (Wang et al., 2015), *Nitrospirota* is associated with N metabolism (Daims et al., 2015), and *Marinimicrobia_SAR406_clade* is associated with the dark fixation of dissolved inorganic carbon (DIC) (Guerrero-Feijóo et al., 2018). The only specific phylum in the male algal bodies was *Elusimicrobiota*, which consists of nitrogen-fixing bacteria (Zheng et al., 2016; Méheust et al., 2020). Interestingly, there were no specific phyla in either male or female receptacles. At the genus level, the results were more striking. For example, the specific genera in male and female algal bodies were still related to metabolism. The specific genera in male algal bodies included the alginate-degrading bacterium *Brachybacterium* (Wang M. et al., 2018), phosphorus-solubilizing bacterium *Psychrobacillus* (Chiba et al., 2022), carotenoid-producing bacterium Flavicella (Teramoto and Nishijima, 2015), and *Rubricoccus* (Nakajima et al., 2017), which contains rhodopsin-producing genes and plays an important role in energy conversion. Interestingly, there were also some gut metabolic bacteria present, such as *Lentisphaera* (related to pentose metabolism) and *Dialister* (Louis et al., 2014) (related to propionic acid production). In addition, the group also included the autotrophic bacterium *Magnetospira* involved in CO_2_ fixation (He, 2014). The number of specific genera in the female algal bodies was much greater than that in the male algal bodies, and their functions were more diverse. However, most of them were involved in nutrient cycling and metabolism, such as *Macrococcus* (Mazhar et al., 2018), with the highest abundance, which can promote carbohydrate and amino acid metabolism; bacteria involved in sulfur metabolism, including *Marinobacterium* (Fuse et al., 2000), *Desulfatitalea* (Pang et al., 2021), *Halanaerobium* (Ravot et al., 2005), and Sva0081_sediment_group (Fan et al., 2018); bacteria involved in nitrogen metabolism, including *Pirellula* (Han et al., 2019), *Costertonia* (Kwon et al., 2006) and *Gemmatimonas* (Park et al., 2017); and complex compound-degrading bacteria, including SM1A02 (Zheng et al., 2020), and *Chryseolinea* (Milkereit et al., 2021). Interestingly, many bacteria have been reported in human and animal intestines, such as *Cloacibacterium* (Nouha et al., 2016), *Prevotella_9*, *Prevotella_2* (Liu Z. et al., 2020), and *Ruminococcaceae_UCG-005* (Gebeyew et al., 2021), which showed the various metabolic capacities of endophytic bacteria specific to female algal bodies. In addition, *Fusobacterium* is an opportunistic anaerobic pathogen (Castellarin et al., 2012), and *Geodermatophilus* has significant antioxidant capacity and can resist multiple environmental stresses (Hongmin et al., 2015). Additionally, there were phototrophic/heterotrophic bacteria of *Candidatus_Actinomarina* that absorb and assimilate dissolved organic matter (DOM) (Xie Z., 2018) and RB41 bacteria important for controlling carbon circulation (Stone et al., 2021).

The differences in specific genera of endophytic bacteria were more apparent in the receptacles. The specific genera with the highest abundance in male receptacles were almost all intestinal bacteria, including *Prevotella_ 1* (Liu Z. et al., 2020), *Erysipelotrichaceae_ UCG-007* (Guo et al., 2020), *Shuttleworthia* (Jin-Shun et al., 2017), *Defluviitaleaceae_U* (Yang et al., 2020), *Faecalibacterium* (Heinken et al., 2014), *Rubellimicrobium* (Xie D., 2018), *Ruminococcus_2* (Ma et al., 2022), *Candidatus_Saccharimonas* (Cao et al., 2022), and *Terrimicrobium* (Qiu et al., 2014). They also included the specific pathogenic bacteria *Peptococcus* (Bourgault et al., 1980) and *Roseomonas* (Rihs et al., 1993). However, in the female receptacles, there were many pathogenic bacteria, such as *Legionella* (Machner and Isberg, 2006), *Mycobacterium* (Palomino, 2009), and *Kocuria* (Basaglia et al., 2002). The bacterial genus *Promicromonospora* specific to the female receptacles *also has* antagonistic effects against *Fusarium oxysporum* and can produce antioxidants. It was particularly interesting that *Steroidobactor* (Fahrbach et al., 2008) was the first known bacteria to grow on estradiol (C-18) and testosterone (C-19), while *Minicystis* (Garcia et al., 2014) could produce steroids. In addition, there were few degrading bacteria in the receptacles of both sexes, and the degrading bacteria in the male receptacles were the phenol-degrading anaerobic bacterium *Thermicanus* (Qi, 2021) and the sulfate-degrading bacterium *Desulfobulbus* (Samain et al., 1986). The female receptacles included the polyethylene degrader *Brevibacillus* (Stone et al., 2021), biopolymer degrader *Tepidisphaera* (Jiang et al., 2020) and various macromolecular degraders such as *Luteimonas* (Lin et al., 2020; Zhou et al., 2021). Moreover, there were alga-soluble bacteria (*Muricauda*) in the male receptacles (Shi et al., 2012), while there were algicidal bacteria [*Kordia* (Sohn et al., 2004) and *Saprospira* (Furusawa et al., 2003)] in the female receptacles.

The results of LEfSe analysis also showed that the genera with high indicator values were different between the sexes and were mainly related to metabolism. For example, *Planctomicrobium*, the bacterium with the highest indicator value in male algal bodies, can participate in the degradation of biopolymers in plant and fungal cell walls (Kulichevskaya et al., 2019), and the bacteria with high indicator values in male receptacles included the degrading bacterium *Rhodococcus* (Larkin et al., 2005) and endophytic nitrogen-fixing bacterium *Delftia* (Han, 2004). *Marinobacterium*, with a high indicator value in female algal bodies, participates in the sulfur cycle (Fuse et al., 2000), and *Coraliomargarita*, with a high indicator value in female receptacles, has been reported as a specific microfloral member in the gut of *Apostichopus japonicus* (Quan et al., 2019).

In summary, there were significant differences in the composition of endophytic bacteria between males and females, mainly associated with material metabolism, as well as the degradation of pollutants, pathogen resistance and antioxidant activity. Studies on higher plants have shown significant differences in enzyme activity, secondary metabolites and endogenous hormone levels between male and female individuals and reported that estrogen is a unique secreted hormone in various brown algae (Li et al., 2006) and that the content of bromophenols in the reproductive tissues of the red alga *Neorhodomela larix* varies between the sexes (Carlson et al., 1989). This indicates that the differences in endophytic bacterial communities mentioned above may mainly be due to differences in material metabolism within the host algae and reproductive tissues of different sexes. However, further confirmation based on combining the metabolomic and transcriptome differences between the sexes of *S. thunbergii* and their receptacles is needed.

### 4.3 The role of host sex in the assembly of the endophytic bacterial community in *S. thunbergia*

The results of the two methods in this study revealed differences in the structure and function of endophytic bacterial communities between male and female *S. thunbergii* and their receptacles. The β-diversity analysis revealed that endophytic bacterial communities in samples from different sexes could be clustered separately. Moreover, the clustering between male and female receptacles was more obvious, indicating that the sex differences in endophytic bacterial communities from receptacles were greater than those from algal bodies. This can be explained by the fact that the differences in the male and female algal bodies of *S. thunbergii* were smaller than those in their receptacles. The smaller difference in endophytic bacterial communities between male and female algal bodies means that the bacterial community in algal bodies of different sexes was more stable than that in the receptacles, but the significant differences in β-diversity between groups indicated that the sex of *S. thunbergii* has a certain impact on the structure of the endophytic bacterial communities in *S. thunbergii* and that the impact was stronger on the endophytic bacterial communities in the receptacles.

The α-diversity results also showed that the abundance and evenness of endophytic bacteria in *S. thunbergii* were higher in females than in males. Previous studies have found that the reproductive tissues of brown macroalgae can secrete unique estrogenic hormones, which may correspond to their metabolic bacteria. Additionally, this study showed the bacterial genera *Steroidobactors* (Fahrbach et al., 2008) that can degrade sex hormones and *Minicystis* (Garcia et al., 2014) that can produce steroids in female receptacles, indicating that the sex of the host can directly affect the composition of endophytic bacteria in the receptacles of *S. thunbergii*.

Moreover, the results of predicted gene function indicated that the difference in the abundance of functional genes between male and female algal bodies was less obvious than that between male and female receptacles. This further confirmed that the differences in bacterial communities of endophytic bacteria between different sexes were mainly due to differences in the receptacles. These differences were mainly related to metabolic genes, which suggested that metabolic differences were the main reason for the differences in endophytic bacteria between male and female algal bodies and receptacles. In addition, the abundance of predicted genes with significant differences was basically the same in both male and female algal bodies but lower than that in the receptacles, and the abundance of predicted genes in the female receptacles was much higher than that in the male receptacles. Many studies have shown significant differences in the content of various chemicals in male and female flowers in plants (Quan et al., 2019; Sowndhararajan et al., 2020). Although algae do not have floral organs, studies have shown that the content of bromophenols in the reproductive structures of the two sexes varies in the red alga *Neorhodomela larix* (Carlson et al., 1989). In this study, it was also found that both *Sva0996_marine_group*, which is involved in the utilization of organic matter (Wang Y. et al., 2018), and *Phormidesmis*, which is related to organophosphorus decomposition, were more abundant in female receptacles. It can be speculated that the differences in the chemicals between male and female receptacles lead to differences in endophytic bacterial communities closely related to host metabolism in the receptacles of different sexes. However, few studies have focused on the differences between male and female algae and their receptacles, and further studies will need to analyze the metabolic differences between algae and receptacles of different sexes and their correlation with endophytic bacterial communities to reveal the mechanism by which the sex of dioecious macroalgae affects the assembly of the endophytic bacterial community.

## 5 Conclusion

In this study, the endophytic bacterial community structures of male and female *S. thunbergii* and their receptacles were compared based on a culture method and high-throughput sequencing. The results of both methods showed that the majority of endophytic bacteria in the two sexes of *S. thunbergii* and their receptacles were the same, but the diversity, abundance of dominant taxa, specific bacterial taxa, and biomarkers differed between the sexes, especially in the samples from receptacles. There was a significant difference in predicted functional abundance between male and female samples, and most of the functions were related to metabolism. It was found for the first time that the sex of the host alga contributes to the community assembly of endophytic bacteria in *S. thunbergii*, and the impact on the endophytic bacterial community was greater in the receptacles. Moreover, many endophytic bacterial strains were obtained, providing experimental materials for the effective utilization and development of algal microbial resources. The results of this study help elucidate the mechanism of endophytic bacterial community assembly in dioecious marine macroalgae and further the understanding of the interaction between endophytic bacteria and macroalgae.

## Data availability statement

The original contributions presented in the study are publicly available. The bacterial sequences obtained in this study have been deposited to National Center for Biotechnology Information (NCBI) under BioProject accession numbers PRJNA830307 and PRJNA830329.

## Author contributions

YZ: Data curation, Formal Analysis, Investigation, Writing – original draft. TS: Data curation, Formal Analysis, Investigation, Writing – original draft. YL: Data curation, Formal Analysis, Investigation, Writing – review and editing. ZY: Data curation, Formal Analysis, Investigation, Writing – review and editing. JC: Writing – review and editing. JW: Writing – review and editing. XY: Writing – review and editing. XT: Funding acquisition, Project administration, Supervision, Writing – review and editing. HX: Supervision, Writing – review and editing.

## Footnotes

1
www.ezbiocloud.net

## References

[B1] Afzal I. Shinwari Z. K. Sikandar S. Shahzad S. (2019). Plant beneficial endophytic bacteria: mechanisms, diversity, host range and genetic determinants. *Microbiol. Res.* 221 36–49. 30825940 10.1016/j.micres.2019.02.001

[B2] Amini Hajiabadi A. Mosleh Arani A. Ghasemi S. Rad M. H. Etesami H. Shabazi Manshadi S. (2021). Mining the rhizosphere of halophytic rangeland plants for halotolerant bacteria to improve growth and yield of salinity-stressed wheat. *Plant Physiol. Biochem.* 163 139–153. 33845330 10.1016/j.plaphy.2021.03.059

[B3] Ammar E. M. Ellogheshtawy H. S. Elloghatoury E. H. Amer S. K. (2021). Green synthesis of polyhydroxyalkanoates polymer by *bacillus iocasae*. *Poly. Int.* 70 1478–1485.

[B4] Amna Xia Y. Farooq M. A. Javed M. T. Kamran M. A. Mukhtar T. (2020). Multi-stress tolerant PGPR *Bacillus xiamenensis* PM14 activating sugarcane (*Saccharum officinarum* L.) red rot disease resistance. *Plant Physiol. Biochem.* 151 640–649. 10.1016/j.plaphy.2020.04.016 32339911

[B5] Amri M. Rjeibi M. R. Gatrouni M. Mateus D. M. R. Asses N. Pinho H. J. O. (2023). Isolation, identification, and characterization of phosphate-solubilizing bacteria from tunisian soils. *Microorganisms* 11:783. 10.3390/microorganisms11030783 36985356 PMC10052640

[B6] Baker S. C. Ferguson S. J. Ludwig B. Page M. D. Richter O. M. van Spanning R. J. (1998). Molecular genetics of the genus *Paracoccus*: metabolically versatile bacteria with bioenergetic flexibility. *Microbiol. Mol. Biol. Rev.* 62 1046–1078. 10.1128/MMBR.62.4.1046-1078.1998 9841665 PMC98939

[B7] Basaglia G. Carretto E. Barbarini D. Moras L. Scalone S. Marone P. (2002). Catheter-related bacteremia due to *Kocuria kristinae* in a patient with ovarian cancer. *J. Clin. Microbiol.* 40 311–313. 10.1128/JCM.40.1.311-313.2002 11773142 PMC120093

[B8] Bourgault A. M. Rosenblatt J. E. Fitzgerald R. H. (1980). *Peptococcus magnus*: a significant human pathogen. *Ann. Intern. Med.* 93 244–248. 10.7326/0003-4819-93-2-244 7406374

[B9] Cao C. Wang L. Ai C. Gong G. Wang Z. Huang L. (2022). Impact of *Lycium barbarum arabinogalactan* on the fecal metabolome in a DSS-induced chronic colitis mouse model. *Food Funct.* 13 8703–8716. 10.1039/d2fo01283a 35912853

[B10] Carlson D. J. Lubchenco J. Sparrow M. A. Trowbridge C. D. (1989). Fine-scale variability of lanosol and its disulfate ester in the temperate red alga *Neorhodomela larix*. *J. Chem. Ecol.* 15 1321–1333. 10.1007/BF01014833 24272015

[B11] Castellarin M. Warren R. L. Freeman J. D. Dreolini L. Krzywinski M. Strauss J. (2012). *Fusobacterium nucleatum* infection is prevalent in human colorectal carcinoma. *Genome Res.* 22 299–306. 10.1101/gr.126516.111 22009989 PMC3266037

[B12] Cerritos R. Vinuesa P. Eguiarte L. E. Herrera-Estrella L. Alcaraz-Peraza L. D. Arvizu-Gómez J. L. (2008). *Bacillus coahuilensis* sp. nov., a moderately halophilic species from a desiccation lagoon in the Cuatro Ciénegas Valley in Coahuila. *Mexico. Int. J. Syst. Evol. Microbiol.* 58(Pt 4), 919–923. 10.1099/ijs.0.64959-0 18398195

[B13] Chandna P. Mayilraj S. Kuhad R. C. (2016). *Bacillus pseudoflexus* sp. nov., a moderately halophilic bacterium isolated from compost. *Ann. Microbiol.* 66 895–905.

[B14] Chen H. Zhao L. Huang X. He J. Huang Y. Zhu L. (2020). Comparison on leaf physiological properties of male and female *Podocarpus macrophyllus*. *GuangXi For. Sci.* 49 125–129.

[B15] Chen L. Wang G. Bu T. Zhang Y. Liu M. Zhang J. (2010). Identification of a moderately halophilic bacterium whb45 and screening of its antimicrobial and antitumor activity. *Microbiol. Chin.* 37 85–90.

[B16] Chiba A. Peine M. Kublik S. Baum C. Schloter M. Schulz S. (2022). Complete genome sequence of *Psychrobacillus* sp. Strain INOP01, a phosphate-solubilizing bacterium isolated from an agricultural soil in Germany. *Microbiol. Resour. Announc.* 11:e0020722. 10.1128/mra.00207-22 35377163 PMC9022493

[B17] Crapart S. Fardeau M. L. Cayol J. Thomas P. Sery C. Ollivier B. (2007). *Exiguobacterium profundum* sp. nov., a moderately thermophilic, lactic acid-producing bacterium isolated from a deep-sea hydrothermal vent. *Int. J. Syst. Evol. Microbiol.* 57 287–292. 10.1099/ijs.0.64639-0 17267965

[B18] Daims H. Lebedeva E. V. Pjevac P. Han P. Herbold C. Albertsen M. (2015). Complete nitrification by *Nitrospira* bacteria. *Nature* 528 504–509. 26610024 10.1038/nature16461 PMC5152751

[B19] Daroonpunt R. Yiamsombut S. Sitdhipol J. Tanasupawat S. (2019). *Bacillus salacetis* sp. nov., a slightly halophilic bacterium from Thai shrimp paste (Ka-pi). *Int. J. Syst. Evol. Microbiol.* 69 1162–1168. 10.1099/ijsem.0.003286 30767851

[B20] Dartora J. Guimarães V. F. Menezes C. R. J. Freiberger M. B. Castoldi G. Gonçalves D. V. (2016). Maize response to inoculation with strains of plant growth-promoting bactéria. *Rev. Bras. Eng. Agríc. Ambient.* 20 606–611.

[B21] Dastogeer K. M. G. Li H. Sivasithamparam K. Jones M. G. K. Wylie S. J. (2018). Host specificity of endophytic mycobiota of wild nicotiana plants from arid regions of northern Australia. *Microbiol. Ecol.* 75 74–87. 10.1007/s00248-017-1020-0 28702707

[B22] de Souza Valente C. Wan A. H. L. (2021). Vibrio and major commercially important vibriosis diseases in decapod crustaceans. *J. Invertebr. Pathol.* 181:107527. 10.1016/j.jip.2020.107527 33406397

[B23] Fadiji A. E. Ayangbenro A. S. Babalola O. O. (2020). Metagenomic profiling of the community structure, diversity, and nutrient pathways of bacterial endophytes in maize plant. *Antonie Van Leeuwenhoek* 113 1559–1571. 10.1007/s10482-020-01463-w 32803452

[B24] Fahrbach M. Kuever J. Remesch M. Huber B. E. Kämpfer P. Dott W. (2008). *Steroidobacter denitrificans* gen. nov., sp. nov., a steroidal hormone-degrading gammaproteobacterium. *Int. J. Syst. Evol. Microbiol.* 58 2215–2223. 10.1099/ijs.0.65342-0 18768632

[B25] Falkenberg F. Voß L. Bott M. Bongaerts J. Siegert P. (2023). New robust subtilisins from halotolerant and halophilic *Bacillaceae*. *Appl. Microbiol. Biotechnol.* 107 3939–3954. 10.1007/s00253-023-12553-w 37160606 PMC10238314

[B26] Fan X. Ding S. Gong M. Chen M. Gao S. Jin Z. (2018). Different influences of bacterial communities on Fe (III) reduction and phosphorus availability in sediments of the *Cyanobacteria*- and macrophyte-dominated zones. *Front. Microbiol.* 9:2636. 10.3389/fmicb.2018.02636 30487778 PMC6247781

[B27] Feng S. Xie G. Liu N. Xu C. Lu F. Feng X. (2020). Isolation and antioxidative activities of algal endophytes. *J. Food Biotechnol.* 39 99–105.

[B28] Feng X. Wang Z. Li X. Wang W. Gu A. Liu Y. (2023). Analysis of endophytic bacterial diversity in rice seeds with regional characteristics in Yunnan Province, China, based on high-throughput sequencing technology. *Curr. Microbiol.* 80:287. 10.1007/s00284-023-03399-6 37458830

[B29] Furusawa G. Yoshikawa T. Yasuda A. Sakata T. (2003). Algicidal activity and gliding motility of *Saprospira* sp. SS98-5. *Can. J. Microbiol.* 49 92–100. 10.1139/w03-017 12718397

[B30] Fuse H. Takimura O. Murakami K. Yamaoka Y. Omori T. (2000). Utilization of dimethyl sulfide as a sulfur source with the aid of light by *Marinobacterium* sp.strain DMS-S1. *Appl. Environ. Microbiol.* 66 5527–5532. 10.1128/AEM.66.12.5527-5532.2000 11097944 PMC92498

[B31] Garcia R. Gemperlein K. Müller R. (2014). *Minicystis rosea* gen. nov., sp. nov., a polyunsaturated fatty acid-rich and steroid-producing soil myxobacterium. *Int. J. Syst. Evol. Microbiol.* 64 3733–3742. 10.1099/ijs.0.068270-0 25114157

[B32] Ge L. Huang Y. Xue J. Qi Q. Zhang Q. Xu X. (2021). Dynamic changes of endogenous hormones in female and male flower of *Taxus cuspidata* during flower development. *J. Beihua Univer.* 22 456–461.

[B33] Gebeyew K. Chen K. Wassie T. Azad M. A. K. He J. Jiang W. (2021). Dietary amylose/amylopectin ratio modulates cecal microbiota and metabolites in weaned goats. *Front. Nutr.* 8:774766. 10.3389/fnut.2021.774766 34957184 PMC8697430

[B34] Ghaderiardakani F. Califano G. Mohr J. Abreu M. Coates J. Wichard T. (2019). Analysis of algal growth- and morphogenesis-promoting bacterial factors (AGPFs) in an integrated multi-trophic aquaculture system for farming the green seaweed Ulva. *Aquacult. Environ. Interactions* 11 375–391.

[B35] Green-Ruiz C. Rodriguez-Tirado V. Gomez-Gil B. (2008). Cadmium and zinc removal from aqueous solutions by *Bacillus jeotgali*: pH, salinity and temperature effects. *Bioresour. Technol.* 99 3864–3870. 10.1016/j.biortech.2007.06.047 17697774

[B36] Guerrero-Feijóo E. Sintes E. Herndl G. J. Varela M. M. (2018). High dark inorganic carbon fixation rates by specific microbial groups in the Atlantic off the Galician coast (NW Iberian margin). *Environ. Microbiol.* 20 602–611. 10.1111/1462-2920.13984 29124858

[B37] Guo J. Zhang X. Saiganesh A. Peacock C. Chen S. Dykes G. A. (2020). Linking the westernised oropharyngeal microbiome to the immune response in Chinese immigrants. *Allergy Asthma Clin. Immunol.* 16:67. 10.1186/s13223-020-00465-7 32944027 PMC7491349

[B38] Gupta R. S. Patel S. Saini N. Chen S. (2020). Robust demarcation of 17 distinct *Bacillus* species clades, proposed as novel *Bacillaceae* genera, by phylogenomics and comparative genomic analyses: description of *Robertmurraya kyonggiensis* sp. nov. and proposal for an emended genus *Bacillus* limiting it only to the members of the subtilis and cereus clades of species. *Int. J. Syst. Evol. Microbiol.* 70 5753–5798. 10.1099/ijsem.0.004475 33112222

[B39] Han J. (2004). *The Studies on the Identification and Biological Characteristics of a Novel rice Endophytic Bacteria Strain Delftia tsuruhatensis HR4 with N2-Fixing Activity.* Doctor’s Thesis. China: Capital Normal University.

[B40] Han W. Zhao S. Liu H. Wu Z. Gu Q. Li Y. (2012). Isolation, identification and agarose degradation of a polysaccharide-degrading marine bacterium *Persicobacter* sp. JZB09. *Microbiol. Chin.* 52 776–783. 22934359

[B41] Han X. Feng J. Zhang L. Cui B. Zhang J. Pan L. (2019). Micro-polluted water treatment by biological contact oxidation process: aeration mode and bacteria community analysis. *Environ. Eng. Sci.* 36 1491–1502.

[B42] He P. Xu S. Huang X. Seswita-Zilda D. Zhang Q. (2012). Isolation and identification of microorganisms from high temperature seaweed beds and study on the characteristics of thermo and salt tolerances. *Microbiol. China* 39 1769–1777.

[B43] He Q. (2014). *Autotrophic Bacteria and Their Community in the Pearl River.* Master’s Thesis. Guangzhou: South China University of Technology.

[B44] Heinken A. Khan M. T. Paglia G. Rodionov D. A. Harmsen H. J. M. Thiele I. (2014). Functional metabolic map of *Faecalibacterium prausnitzii*, a beneficial human gut microbe. *J. Bacteriol.* 196 3289–3302. 10.1128/JB.01780-14 25002542 PMC4135701

[B45] Ho X. Y. Katermeran N. P. Deignan L. K. Phyo M. Y. Ong J. F. M. Goh J. X. (2021). Assessing the diversity and biomedical potential of microbes associated with the neptune’s cup sponge *Cliona patera*. *Front. Microbiol.* 12:631445. 10.3389/fmicb.2021.631445 34267732 PMC8277423

[B46] Hollants J. Leroux O. Leliaert F. Decleyre H. De Clerck O. Willems A. (2011). Who is in there? exploration of endophytic bacteria within the siphonous green seaweed *Bryopsis* (*Bryopsidales, Chlorophyta*). *PLoS One* 6:e26458. 10.1371/journal.pone.0026458 22028882 PMC3196581

[B47] Huo X. Wang Y. Zhang D. Gao T. Liu M. (2020). Characteristics and diversity of endophytic bacteria in endangered Chinese herb *Glehnia littoralis* based on illumina sequencing. *Pol. J. Microbiol.* 69 283–291. 10.33073/pjm-2020-031 33574857 PMC7810123

[B48] Ivanova E. P. Alexeeva Y. A. Zhukova N. V. Gorshkova N. M. Buljan V. Nicolau D. V. (2004). Bacillus algicola sp. nov., a novel filamentous organism isolated from brown alga *Fucus evanescens*. *Syst. Appl. Microbiol.* 27 301–307. 10.1078/0723-2020-00269 15214635

[B49] Jiang B. Zeng Q. Liu J. Hou Y. Xu J. Li H. (2020). Enhanced treatment performance of phenol wastewater and membrane antifouling by biochar-assisted EMBR. *Bioresour. Technol.* 306:123147. 10.1016/j.biortech.2020.123147 32171174

[B50] Jin-Shun Z. Cai-Xia W. U. Ming-Mei L. I. U. Xiao-Shuang S. U. Kang Z. Guo-Qi Z. (2017). Effects of alfalfa flavonoids as dietary additives on bacterial flora in the rumen of dairy cows. *Acta Pratacult. Sinica* 26:82.

[B51] Kang C. L. Li Q. F. Zhang Y. Chen S. B. Wang Y. (2018). Purifying effect of three heterotrophic nitrification-aerobic denitrification bacteria strains on the farming water of verasper variegates. *Prog. Fishery Sci.* 39 42–48.

[B52] Kulichevskaya I. S. Naumoff D. G. Ivanova A. A. Rakitin A. L. Dedysh S. N. (2019). Detection of chitinolytic capabilities in the freshwater planctomycete *planctomicrobium piriforme*. *Microbiology* 88 423–432.

[B53] Larkin M. J. Kulakov L. A. Allen C. C. R. (2005). Biodegradation and rhodococcus–masters of catabolic versatility. *Curr. Opin. Biotechnol.* 16 282–290. 10.1016/j.copbio.2005.04.007 15961029

[B54] Li J. Luo C. Song M. Dai Q. Jiang L. Zhang D. (2017). Biodegradation of phenanthrene in polycyclic aromatic hydrocarbon-contaminated wastewater revealed by coupling cultivation-dependent and -independent approaches. *Environ. Sci. Technol.* 51 3391–3401. 10.1021/acs.est.6b04366 28181806

[B55] Li L. Lei G. Li L. Du Z. Zhen J. Wang J. (2021). Diversity analysis on endophytic bacterial community in different organs of *Arachis hypogaea Linn*, based on high-throughput sequencing. *J. Peanut. Sci.* 50 1–20.

[B56] Li W. Li D. Du X. (2006). Advance in male-attractants from *Phaeophyta*. *Mar. Sci.* 30 79–84.

[B57] Li Z. Li J. Wu C. Xu B. Song P. Liu Z. (2022). Total flavonoid content and antioxidant activity of female and male flower buds of *Populus tomentosa*. *Central S. Pharmacy* 20 1034–1038.

[B58] Liaqat F. Eltem R. (2016). Identification and characterization of endophytic bacteria isolated from in vitro cultures of peach and pear rootstocks. *3 Biotech* 6:120. 10.1007/s13205-016-0442-6 28330195 PMC4909027

[B59] Lin P. Yan Z.-F. Li C.-T. (2020). *Luteimonas cellulosilyticus* sp. nov., cellulose-degrading bacterium isolated from soil in changguangxi national wetland park. *China. Curr. Microbiol.* 77 1341–1347. 10.1007/s00284-020-01934-3 32140833

[B60] Ling L. Yang C. Li Z. Luo H. Feng S. Zhao Y. (2022). Plant endophytic bacteria: a potential resource pool of electroactive micro-organisms. *J. Appl. Microbiol.* 132, 2054–2066. 10.1111/jam.15368 34796592

[B61] Liu L. Lu L. Li H. Meng Z. Dong T. Peng C. (2021). Divergence of phyllosphere microbial communities between females and males of the dioecious *Populus cathayana*. *Mol. Plant Microbe Interact.* 34 351–361. 10.1094/MPMI-07-20-0178-R 33290085

[B62] Liu Y. Xu P. Yang F. Li M. Yan H. Li N. (2019). Composition and diversity of endophytic bacterial community in seeds of super hybrid rice ‘Shenliangyou 5814’ (*Oryza sativa* L.) and its parental lines. *Plant Growth Regul.* 87 257–266.

[B63] Liu Y. Yan H. Zhang X. Zhang R. Li M. Xu T. (2020). Investigating the endophytic bacterial diversity and community structures in seeds of genetically related maize (*Zea mays* L.) genotypes. *3 Biotech* 10:27. 10.1007/s13205-019-2034-8 31950006 PMC6942555

[B64] Liu Z. Guo F. Zhang B. Xaing D. Zhao Z. Zhao S. (2020). Effects of complex probiotics derived from different variety on enteric duct microbes in duroc×diannian smallear pig. *mLife* 40 21–29.

[B65] Louis P. Hold G. L. Flint H. J. (2014). The gut microbiota, bacterial metabolites and colorectal cancer. *Nat. Rev. Microbiol.* 12 661–672. 25198138 10.1038/nrmicro3344

[B66] Lu J. Chen Y. Yin T. (2021). Research progress on sex determination genes of woody plants. *Chin. Bull. Bot.* 56:90.

[B67] Ma J. Zhang Y. Dong C. Chai Y. Guo Q. (2022). Comparison of the chemical constituents and in vitro hypoglycemic effect of *Polygonatum* from different origins. *Modern Food Sci. Technol.* 38 116–126.

[B68] Machner M. P. Isberg R. R. (2006). Targeting of host Rab GTPase function by the intravacuolar pathogen *Legionella pneumophila*. *Dev. Cell* 11 47–56. 10.1016/j.devcel.2006.05.013 16824952

[B69] Mahmood A. Takagi K. Ito K. Kataoka R. (2019). Changes in endophytic bacterial communities during different growth stages of cucumber (*Cucumis sativus* L.). *World J. Microbiol. Biotechnol.* 35:104. 10.1007/s11274-019-2676-z 31236765

[B70] Marag P. S. Suman A. (2018). Growth stage and tissue specific colonization of endophytic bacteria having plant growth promoting traits in hybrid and composite maize (*Zea mays* L.). *Microbiol. Res.* 214 101–113. 10.1016/j.micres.2018.05.016 30031472

[B71] Mazhar S. Hill C. McAuliffe O. (2018). The genus *Macrococcus*: an insight into its biology, evolution, and relationship with *Staphylococcus*. *Adv. Appl. Microbiol.* 105 1–50. 10.1016/bs.aambs.2018.05.002 30342720

[B72] Méheust R. Castelle C. J. Matheus Carnevali P. B. Farag I. F. He C. Chen L.-X. (2020). Groundwater *Elusimicrobia* are metabolically diverse compared to gut microbiome *Elusimicrobia* and some have a novel nitrogenase paralog. *ISME J.* 14 2907–2922. 10.1038/s41396-020-0716-1 32681159 PMC7785019

[B73] Mei X. (2019). *Community Structures and Functions of Bacteria Associated with Blooming Seaweeds in the Yellow Sea.* Master’s Thesis. China: University of Chinese Academy of Sciences.

[B74] Mei Y. Shayimu G. Zhi D. Jing Z. Xiao J. Qi Y. (2021). Diversity and function analysis of endophytic bacterial community in different tissues of *Lycium ruthenicum* Murr. *Acta Microbiol. Sin.* 61 152–166.

[B75] Milkereit J. Geisseler D. Lazicki P. Settles M. L. Durbin-Johnson B. P. Hodson A. (2021). Interactions between nitrogen availability, bacterial communities, and nematode indicators of soil food web function in response to organic amendments. *Appl. Soil Ecol.* 157:103767.

[B76] Moradi M. Song Z. Xiao T. (2018). Exopolysaccharide produced by *Vibrio neocaledonicus* sp. as a green corrosion inhibitor: production and structural characterization. *J. Mater. Sci. Technol.* 34 2447–2457.

[B77] Munir S. Li Y. He P. Huang M. He P. He P. (2020). Core endophyte communities of different citrus varieties from citrus growing regions in China. *Sci. Rep.* 10:3648. 10.1038/s41598-020-60350-6 32108149 PMC7046616

[B78] Nakajima Y. Yoshizawa S. Park S. Kumagai Y. Wong S.-K. Ogura Y. (2017). Draft genome sequence of *Rubricoccus marinus* SG-29T, a marine bacterium within the family *Rhodothermaceae*, which contains two different Rhodopsin genes. *Genome Announc.* 5:e00990-17. 10.1128/genomeA.00990-17 28935744 PMC5609423

[B79] Nedashkovskaya O. I. Van Trappen S. Frolova G. M. De Vos P. (2012). Bacillus berkeleyi sp. nov., isolated from the sea urchin *Strongylocentrotus intermedius*. *Arch. Microbiol.* 194 215–221. 10.1007/s00203-011-0771-0 22102083

[B80] Nikolaeva E. V. Usov A. I. Sinitsyn A. P. Tambiev A. H. (1999). Degradation of agarophytic red algal cell wall components by new crude enzyme preparations. *J. Appl. Phycol.* 11 385–389.

[B81] Nouha K. Kumar R. S. Tyagi R. D. (2016). Heavy metals removal from wastewater using extracellular polymeric substances produced by *Cloacibacterium normanense* in wastewater sludge supplemented with crude glycerol and study of extracellular polymeric substances extraction by different methods. *Bioresour. Technol.* 212 120–129. 10.1016/j.biortech.2016.04.021 27089427

[B82] Orsi W. D. Smith J. M. Liu S. Liu Z. Sakamoto C. M. Wilken S. (2016). Diverse, uncultivated bacteria and archaea underlying the cycling of dissolved protein in the ocean. *ISME J.* 10 2158–2173. 10.1038/ismej.2016.20 26953597 PMC4989311

[B83] Palomino J. C. (2009). Molecular detection, identification and drug resistance detection in *Mycobacterium tuberculosis*. *FEMS Immunol. Med. Microbiol.* 56 103–111. 10.1111/j.1574-695X.2009.00555.x 19416361

[B84] Pang Y. Wang J. Li S. Ji G. (2021). Long-term sulfide input enhances chemoautotrophic denitrification rather than DNRA in freshwater lake sediments. *Environ. Pollut.* 270:116201. 10.1016/j.envpol.2020.116201 33321438

[B85] Park D. Kim H. Yoon S. (2017). Nitrous oxide reduction by an obligate aerobic bacterium, *Gemmatimonas aurantiaca* Strain T-27. *Appl. Environ. Microbiol.* 83:e00502-17. 10.1128/AEM.00502-17 28389533 PMC5452805

[B86] Patel S. Gupta R. (2020). A phylogenomic and comparative genomic framework for resolving the polyphyly of the genus *Bacillus*: proposal for six new genera of *Bacillus* species, *Peribacillus* gen. nov., *Cytobacillus* gen. nov., *Mesobacillus* gen. nov., *Neobacillus* gen. nov., *Metabacillus* gen. nov. and *Alkalihalobacillus* gen. nov. *Int. J. Syst. Evol. Microbiol.* 70 406–438. 10.1099/ijsem.0.003775 31617837

[B87] Pramanic A. Sharma S. Dhanorkar M. Prakash O. Singh P. (2023). Endophytic microbiota of floating aquatic plants: recent developments and environmental prospects. *World J. Microbiol. Biotechnol.* 39:96. 10.1007/s11274-023-03543-1 36765023

[B88] Qi Y. (2021). *Study on the Degradation of Phenolic Pollutants in Coal Chemical Wastewater by Fenton-Like and Anaerobic Digestion Mythod.* Master’s Thesis. Qingdao: Qingdao University of Science and Technology.

[B89] Qiu Y.-L. Kuang X.-Z. Shi X.-S. Yuan X.-Z. Guo R.-B. (2014). *Terrimicrobium sacchariphilum* gen. nov., sp. nov., an anaerobic bacterium of the class “*Spartobacteria*” in the phylum *Verrucomicrobia*, isolated from a rice paddy field. *Int. J. Syst. Evol. Microbiol.* 64 1718–1723. 10.1099/ijs.0.060244-0 24535138

[B90] Quan Z. Zhang P. Zhang Y. Wang L. Ding J. Chang Y. (2019). Bacterial community and function in the intestinal tracts of sea cucumber (*Apostichopus japonicus*) at different temperatures. *Chinese J. Ecol.* 38 2756–2764.

[B91] Ravot G. Casalot L. Ollivier B. Loison G. Magot M. (2005). rdlA, a new gene encoding a rhodanese-like protein in *Halanaerobium congolense* and other thiosulfate-reducing anaerobes. *Res. Microbiol.* 156 1031–1038. 10.1016/j.resmic.2005.05.009 16085393

[B92] Rihs J. D. Brenner D. J. Weaver R. E. Steigerwalt A. G. Hollis D. G. Yu V. L. (1993). *Roseomonas*, a new genus associated with bacteremia and other human infections. *J. Clin. Microbiol.* 31 3275–3283. 10.1128/jcm.31.12.3275-3283.1993 8308122 PMC266400

[B93] Rizzo C. Malavenda R. Gerçe B. Papale M. Syldatk C. Hausmann R. (2019). Effects of a simulated acute oil spillage on bacterial communities from arctic and antarctic marine sediments. *Microorganisms* 7:632. 10.3390/microorganisms7120632 31801240 PMC6956123

[B94] Samain E. Albagnac G. Legall J. (1986). Redox studies of the tetraheme cytochrome c3 isolated from the propionate-oxidizing, sulfate-reducing bacterium *Desulfobulbus elongatus*. *FEBS Lett.* 204 247–250. 10.1016/0014-5793(87)80772-x 3582662

[B95] Sharma A. Kohli P. Singh Y. Schumann P. Lal R. (2016). *Fictibacillus halophilus* sp. nov., from a microbial mat of a hot spring atop the Himalayan Range. *Int J. Syst. Evol. Microbiol.* 66 2409–2416. 10.1099/ijsem.0.001051 27031366

[B96] Shi R. Huang H. Qi Z. Hu W. Tian Z. Dai M. (2012). Algicidal activity against *Prorocentrum micans* by a marine bacterium isolated from a HABs area. *South China. Acta Ecol. Sin.* 32 4993–5001.

[B97] Shi X. G. Li Y. Zheng W. H. Xiao Y. C. Liu L. M. Chen J. F. (2020). Isolation and algicidal characteristics of a specific algicidal bacterium on *Skeletonema costatum*. *Microbiol. China* 47 3527–3538.

[B98] Sohn J. H. Lee J.-H. Yi H. Chun J. Bae K. S. Ahn T.-Y. (2004). *Kordia algicida* gen. nov., sp. nov., an algicidal bacterium isolated from red tide. *Int. J. Syst. Evol. Microbiol.* 54 675–680. 10.1099/ijs.0.02689-0 15143006

[B99] Son H. Lee K. (2023). Genome sequences of *Metabacillus* sp. Strain B2-18, isolated from human skin, and *Metabacillus endolithicus* KCTC 33579T. *Microbiol. Resour. Announc.* 12:e0134822. 10.1128/mra.01348-22 36651753 PMC9933729

[B100] Song F. Li C. Zhang N. He X. Yang H. Yan Z. (2023). *Alkalihalobacillus clausii* PA21 transcriptome profiling and functional analysis revealed the metabolic pathway involved in glycoalkaloids degradation. *Int. J. Biol. Macromol.* 242:124682. 10.1016/j.ijbiomac.2023.124682 37164133

[B101] Song T. Zhang W. Wei C. Jiang T. Xu H. Cao Y. (2015). Isolation and characterization of agar-degrading endophytic bacteria from plants. *Curr. Microbiol.* 70 275–281. 10.1007/s00284-014-0713-6 25331792

[B102] Song X. Li L. Cui G. Ding G. (2020). Population structure analysis of endophytic bacteria in roots of *Platycodon grandiflorum* (Jacq)A. DC with different growth years. *Guangxi Agric. Sci.* 51 2358–2366.

[B103] Sowndhararajan K. Kim J.-H. Song J. E. Kim M. Kim S. (2020). Chemical components of male and female flowers of *Schisandra chinensis*. *Biochem. Syst. Ecol.* 92:104121.

[B104] Stone B. W. Li J. Koch B. J. Blazewicz S. J. Dijkstra P. Hayer M. (2021). Nutrients cause consolidation of soil carbon flux to small proportion of bacterial community. *Nat. Commun.* 12 3381. 10.1038/s41467-021-23676-x PMC8184982 34099669

[B105] Sun H. Tan S. Liang J. Yang G. Xin Y. Zhang X. (2020). Horizontal and vertical distribution of dimethyl sulfoniopropionate (DMSP) producing and catabolizing bacteria in the East China Sea. *Microbiol. Chin.* 60 1865–1881.

[B106] Tang X. (2020). Characteristics and research progress of sex-specific responses to environmental stresses of dioecious plants. *Periodical Ocean Univer. China* 50 74–81.

[B107] Teramoto M. Nishijima M. (2015). *Flavicella marina* gen. nov., sp. nov., a carotenoid-producing bacterium from surface seawater. *Int. J. Syst. Evol. Microbiol.* 65 799–804. 10.1099/ijs.0.000018 25481292

[B108] Verhoeven J. T. P. Kavanagh A. N. Dufour S. C. (2017). Microbiome analysis shows enrichment for specific bacteria in separate anatomical regions of the deep-sea carnivorous sponge *Chondrocladia grandis*. *FEMS Microbiol. Ecol.* 93:fiw214. 10.1093/femsec/fiw214 27756769

[B109] Wang C. Sun Q. Ji Y. N. Liu L. Zhang K. Li J. K. (2023). A high saline-alkali tolerance strain Rossellomorea aquimaris S-2 and its application. *Shandong Agricultural University, CN116948867A*.

[B110] Wang F. Sun X. Li F. (2007). Studies on sexual reproduction and seedling-rearing of *Sargassum thunbergii*. *Mar. Fish. Res.* 27 1–6.

[B111] Wang J. Li Y. Yang Z. Sun T. Yu X. Zhao Y. (2022a). Sex plays a role in the construction of epiphytic bacterial communities on the algal bodies and receptacles of *Sargassum thunbergii*. *Front. Microbiol.* 13:935222. 10.3389/fmicb.2022.935222 35958132 PMC9360977

[B112] Wang J. Yang Z. Wang G. Shang S. Tang X. Xiao H. (2022b). Diversity of epiphytic bacterial communities on male and female *Sargassum thunbergii*. *AMB Express* 12:97. 35841460 10.1186/s13568-022-01439-1 PMC9288574

[B113] Wang M. Wang X. Chen L. (2018). In situ screening of cultivable alginate-degrading microorganism on surface of brown seaweed. *Microbiol. China* 45 1853–1860.

[B114] Wang S. Liu J. Sun J. Jia N. Jin N. Liu J. (2021). Diversity and antibacterial activity of endophytic bacteria in roots and stems of *Dendrobium officinale* with different cultivation patterns. *Microbiol. Chin.* 61 4006–4025.

[B115] Wang X. Sharp C. E. Jones G. M. Grasby S. E. Brady A. L. Dunfield P. F. (2015). Stable-isotope probing identifies uncultured planctomycetes as primary degraders of a complex heteropolysaccharide in soil. *Appl. Environ. Microbiol.* 81 4607–4615. 10.1128/AEM.00055-15 25934620 PMC4551180

[B116] Wang Y. Wang B. Dann L. M. Mitchell J. G. Hu X. Tang H. (2018). Bacterial community structure in the Bohai Strait provides insights into organic matter niche partitioning. *Continental Shelf Res.* 169 46–54.

[B117] Wang Z. (2007). *The Physiological Ecology and Reproduction Biology of Sargassum thunbergii.* Master’s Thesis. China: University of Chinese Academy of Sciences.

[B118] Webster G. Mullins A. J. Cunningham-Oakes E. Renganathan A. Aswathanarayan J. B. Mahenthiralingam E. (2020). Culturable diversity of bacterial endophytes associated with medicinal plants of the Western Ghats, India. *FEMS Microbiol. Ecol.* 96:fiaa147. 10.1093/femsec/fiaa147 32710748 PMC7422900

[B119] Wu Q. Chen D. Zhou W. Zhang X. Ao J. (2022). Long-term fertilization has different impacts on bacterial communities and phosphorus forms in sugarcane rhizosphere and bulk soils under low-P stress. *Front. Plant Sci.* 13:1019042. 10.3389/fpls.2022.1019042 36212295 PMC9539793

[B120] Xie D. (2018). *Research of Synergistic Reaction Between Intestinal Microflora Quorum Sensing Inhibitor and Jiedu Quyu ziyin Recipe on Treating MRL/lpr Mice.* Master’s Thesis. Hangzhou: Zhejiang Chinese Medical University.

[B121] Xie Z. (2018). *The Molecular Biogeochemistry of Marine Organic Matter in the Oligotrophic South China Sea.* Doctor’s Thesis. China: XiaMen University.

[B122] Yang Q. Liang Q. Balakrishnan B. Belobrajdic D. P. Feng Q. J. Zhang W. (2020). Role of dietary nutrients in the modulation of gut microbiota: a narrative review. *Nutrients* 12:381. 10.3390/nu12020381 PMC7071260 32023943

[B123] Yang Z. Chen J. Shang S. Wang J. Xue S. Tang X. (2022). Diversity of epiphytic bacterial communities on male and female *Porphyra haitanensis*. *Ann. Microbiol.* 72 1–8. 35841460

[B124] Yarte M. E. Gismondi M. I. Llorente B. E. Larraburu E. E. (2022). Isolation of endophytic bacteria from the medicinal, forestal and ornamental tree Handroanthus impetiginosus. *Environ. Technol.* 43, 1129–1139. 10.1080/09593330.2020.1818833 32875965

[B125] Yuan J. (2008). *Diversity of PAH-Degrading Bacteria in Deep Water of Indian Ocean, Classification and Degradation Pathway Research of Some Novel Bacteria.* Doctor’s Thesis. China: Xiamen University.

[B126] Zeng S. Zeng S. Liang T. Li L. Xing X. Chen H. (2022). Pollination system and reproductive allocation strategies of dioecious tree *Salix dunnii*. *J. Trop. Subtrop. Bot.* 30 357–366.

[B127] Zhang A. Guo B. Han X. Li X. (2020). Diversity of endophytic bacteria in seeds of *Hippophae rhamnoides* subsp. sinensis in two different habitats. *Acta Ecol. Sin.* 40 5247–5257.

[B128] Zhang A. Yin Y. Kong W. Zhu X. Yang Y. (2021). Diversity of endophytic bacteria in five types of tissues of *Hippophae tibetana*. *Biodiv. Sci.* 29 1236–1244.

[B129] Zhang C. Ma X. Zhu R. Liu Z. Gu M. Zhang J. (2020). Analysis of the endophytic bacteria community structure and function of *Panax notoginseng* based on high-throughput sequencing. *Curr. Microbiol.* 77 2745–2750. 10.1007/s00284-020-02068-2 32506240

[B130] Zhang K. (2017). *Manganese Tolerance and Enhanced Phytoremediation Potential of Endophytic Bacteria in Foxtail algae.* Master’s Thesis. China: Guangxi University.

[B131] Zhang Q. Zhang Q. Huang X. Guo X. (2015). Advance in endophytic bacterial diversity of wetland plants. *Wetland Sci.* 13 233–243.

[B132] Zhang Y. Shi P. Ma J. (2013). *Exiguobacterium* spp. and their applications in environmental remediation. *Chin. J. Appl. Environ. Biol.* 19 898–904.

[B133] Zhang Y. Yang Q. Ling J. Tang X. Zhang W. Dong J. (2021). Complete genome sequence of *Metabacillus* sp. cB07, a bacterium inducing settlement and metamorphosis of coral larvae. *Mar. Genomics* 60:100877. 10.1016/j.margen.2021.100877 34627550

[B134] Zhang Z. Hu L. Liu L. Ji M. (2020). Identification and antibacterial properties of an antagonistic bacterium *Bacillus safensis* against walnut fungal disease. *J. Henan Agric. Sci.* 49 97–104.

[B135] Zhang Z. Y. Zhou J. Yuan L. Gao R. C. (2019). Effects of mixed starter cultures and exogenous L-Lys on fermentation quality of fish paste. *Schl. Food Biol. Eng.* 40 108–114.

[B136] Zheng H. Dietrich C. Radek R. Brune A. (2016). *Endomicrobium proavitum*, the first isolate of *Endomicrobia class*. nov. (phylum *Elusimicrobia*)–an ultramicrobacterium with an unusual cell cycle that fixes nitrogen with a Group IV nitrogenase. *Environ. Microbiol.* 18 191–204. 10.1111/1462-2920.12960 26119974

[B137] Zheng H. Zhang P. Qin J. Guo J. Deng J. (2022). High-throughput sequencing-based analysis of the composition and diversity of endophytic bacteria community in tubers of *Gastrodia elata* f*.glauca*. *Front. Microbiol.* 13:1092552. 10.3389/fmicb.2022.1092552 36733772 PMC9887035

[B138] Zheng Q. Wang Y. Lu J. Lin W. Chen F. Jiao N. (2020). Metagenomic and metaproteomic insights into photoautotrophic and heterotrophic interactions in a *Synechococcus* culture. *mBio* 11:e03261-19. 10.1128/mBio.03261-19 32071270 PMC7029141

[B139] Zhou J. Chen J. Ma J. Xu N. Xin F. Zhang W. (2021). *Luteimonas wenzhouensis* sp. nov., a chitinolytic bacterium isolated from a landfill soil. *Curr. Microbiol.* 78 383–388. 10.1007/s00284-020-02293-9 33258058

