# Supplementary material for: The host sex contributes to the endophytic bacterial community in Sargassum thunbergii and their receptacles
Source: Front Microbiol. 2024 Mar 15;15:1334918. doi: 10.3389/fmicb.2024.1334918 (PMC10978810; doi:10.3389/fmicb.2024.1334918)
Supplement: Supplementary file 1 [file Data_Sheet_1.pdf]

Supplementary Materials

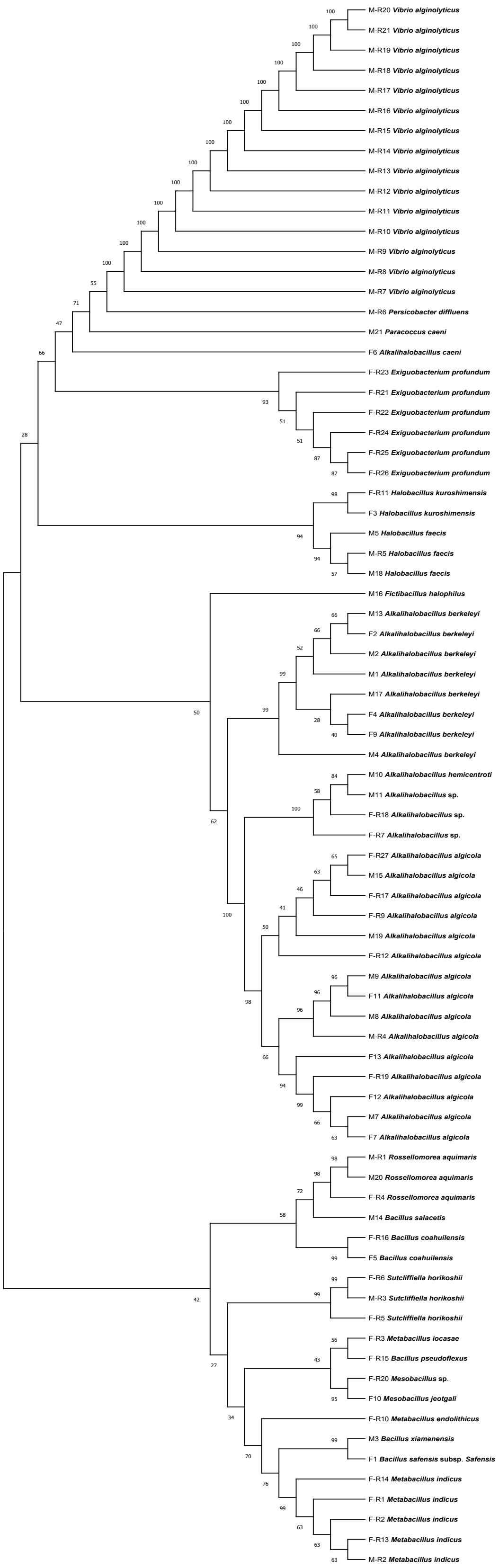

Supplemental Figure 1. Phylogenetic tree of 78 strains of culturable endophytic bacteria isolated from male and female *S. thunbergii* and their receptacles

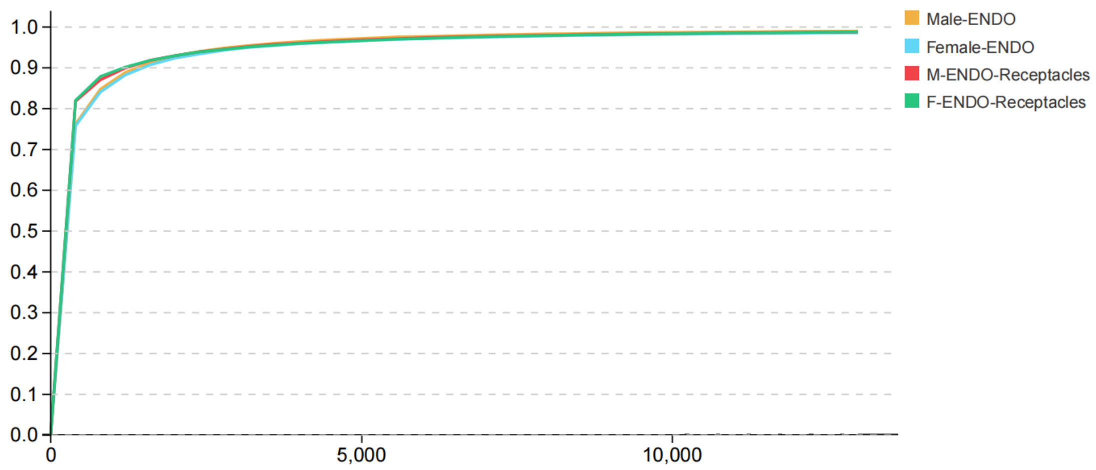

Supplemental Figure 2. Dilution curves of coverage of endophytic bacteria in male and female *S. thunbergii* and their receptacles

Supplemental Table 1A. The function of isolated culturable endophytic bacteria from male and female *S. thunbergia*

phylum	genus	species	Function of species	Male	Female
Bacillota	*Alkalihalobacillus*	*Alkalihalobacillus algicola*	Bacteria with alkali tolerance and alginolytic ability isolated from brown alga (Ivanova, E. P. et al., 2004)	5	4
		Alkalihalobacillus berkeleyi	Saline-alkali tolerant bacteria isolated from sea urchin (Patel S. et al., 2020; Nedashkovskaya, O. I. et al., 2012)	5	3
		Alkalihalobacillus caeni	Saline-alkali tolerant bacteria (Patel S. et al., 2020)	0	1
		Alkalihalobacillus hwajinpoensis	Saline-alkali tolerant bacteria (Patel S. et al.2020); Nitrogen metabolism bacteria (Kang, C. et al., 2018)	1	0
		Alkalihalobacillus sp.	Saline-alkali tolerant bacteria (Patel S. et al., 2020)	1	0
	Rossellomorea	*Rossellomorea aquimaris*	Saline-alkali tolerant bacteria（Wang C,Q. et al., 2023）	1	0
	Bacillus	*Bacillus safensis subsp. Safensis*	Bacteria with high tolerance to salt and ultraviolet light (Amini Hajiabadi, A. et al., 2021)	0	1
		Bacillus coahuilensis	Moderately halophilic bacteria from desiccation lagoon (Cerritos, R. et al., 2018）	0	1
		Bacillus salacetis	Slightly halophilic bacteria (Daroonpunt, R. et al., 2019）	1	0
		Bacillus xiamenensis	Multi-stress tolerant bacteria with disease resistance (Amna, Xia et al., 2020)	1	0
	Fictibacillus	*Fictibacillus halophilus*	Salt-loving bacteria (Sharma, A. et al., 2016)	1	0
	Halobacillus	*Halobacillus faecis*	Moderately halophilic bacteria (Zhang, Z.Y. et al., 2019)	2	0
		Halobacillus kuroshimensis	Halophilic bacteria with capacity of dissolving *skeletonema costatum* (Shi, X.G. et al., 2020)	0	1
	Mesobacillus	*Mesobacillus jeotgali*	Bacteria with biosorption ability of heavy metals (Green-Ruiz, C. et al., 2008)	0	1
Pseudomonadota	*Paracoccus*	*Paracoccus caeni*	Bacteria with multiple metabolic functions (Baker, S. C. et al., 1998)	1	0
		sum		19	12

Note: The numbers in the table represent the number of isolated strains of that species.

Supplemental Table 1B. The function for isolated culturable endophytic bacteria in male and female receptacles of *S. thunbergii*

phylum	genus	Species	Function of species	Male	Female
Bacillota	*Alkalihalobacillus*	*Alkalihalobacillus algicola*	Bacteria with alkali tolerance and alginolytic ability isolated from brown alga (Ivanova, E. P. et al., 2004)	1	5
		Alkalihalobacillus sp.-1	Saline-alkali tolerant bacteria (Patel S. et al., 2020)	0	1
		Alkalihalobacillus sp.-2	Saline-alkali tolerant bacteria (Patel S. et al., 2020)	0	1
	Exiguobacterium	*Exiguobacterium profundum*	Bacteria with adaption to salt and alkali (Zhang, Y. et al., 2013); Moderately thermophilic, lactic acid-producing bacteria (Crapart, S. et al., 2007)	0	6
	Metabacillus	*Metabacillus indicus*	Halotolerant bacteria (Falkenberg, F. et al. 2023)	1	4
		Metabacillus iocasae	PHAs producer (Ammar, E. M. et al., 2021)	0	1
		Metabacillus endolithicus	Bacteria isolated from beach pebbles (Son, H. et al., 2023)	0	1
		Metabacillus sp.	Bacteria inducing settlement and metamorphosis of coral larvae (Zhang, Y. et al., 2021)	0	1
	Sutcliffiella	*Sutcliffiella horikoshii*	Bacteria with adsorption and passivation ability of heavy metals (Gupta, R. S. et al., 2020)	1	2
	Halobacillus	*Halobacillus kuroshimensis*	Halophilic bacteria with capacity of dissolving *skeletonema costatum* (Shi, X.G. et al., 2020)	0	1
		Halobacillus faecis	Moderately halophilic bacteria (Zhang, Z.Y. et al., 2019)	1	0
	Rossellomorea	*Rossellomorea aquimaris*	Saline-alkali tolerant bacteria (Wang C, Q. et al.,2023)	1	1
	Bacillus	*Bacillus pseudoflexus*	Moderately halophilic bacteria isolated from compost (Chandna, P. et al., 2016)	0	1
		Bacillus coahuilensis	Moderately halophilic bacteria from desiccation lagoon (Cerritos, R. et al., 2018)	0	1
Pseudomonadota	*Vibrio*	*Vibrio alginolyticus*	Alginic acid-dissolving bacteria (de Souza Valente. et al., 2021).	15	0
Bacteroidota	*Persicobacter*	*Persicobacter diffluens*	Agarolytic bacteria (Nikolaeva, E. V. et al., 1999)	1	0
		sum		21	26

Note: The numbers in the table represent the number of isolated strains of that species.
